# Genetic evidence supports linguistic affinity of Mlabri - a hunter-gatherer group in Thailand

**DOI:** 10.1186/1471-2156-11-18

**Published:** 2010-03-19

**Authors:** Shuhua Xu, Daoroong Kangwanpong, Mark Seielstad, Metawee Srikummool, Jatupol Kampuansai, Li Jin, The HUGO Pan-Asian SNP Consortium*

**Affiliations:** 1 Chinese Academy of Sciences and Max Planck Society (CAS-MPG) Partner Institute for Computational Biology, Shanghai Institutes for Biological Sciences, Chinese Academy of Sciences, Shanghai 200031, China; 2 Key Laboratory of Computational Biology, CAS-MPG Partner Institute for Computational Biology, Chinese Academy of Sciences, Shanghai 200031, China; 3 Department of Biology, Faculty of Science, Chiang Mai University, 239 Huay Kaew Road, Chiang Mai 50202, Thailand; 4 Genome Institute of Singapore, 60 Biopolis Street #02-01, Genome, 138672, Singapore; 5 State Key Laboratory of Genetic Engineering and Ministry of Education Key Laboratory of Contemporary Anthropology, School of Life Sciences and Institutes of Biomedical Sciences, Fudan University, Shanghai 200433, China; 6 China Medical City (CMC) Institute of Health Sciences, Taizhou, Jiangsu 225300, China

## Abstract

**Background:**

The Mlabri are a group of nomadic hunter-gatherers inhabiting the rural highlands of Thailand. Little is known about the origins of the Mlabri and linguistic evidence suggests that the present-day Mlabri language most likely arose from Tin, a Khmuic language in the Austro-Asiatic language family. This study aims to examine whether the genetic affinity of the Mlabri is consistent with this linguistic relationship, and to further explore the origins of this enigmatic population.

**Results:**

We conducted a genome-wide analysis of genetic variation using more than fifty thousand single nucleotide polymorphisms (SNPs) typed in thirteen population samples from Thailand, including the Mlabri, Htin and neighboring populations of the Northern Highlands, speaking Austro-Asiatic, Tai-Kadai and Hmong-Mien languages. The Mlabri population showed higher LD and lower haplotype diversity when compared with its neighboring populations. Both model-free and Bayesian model-based clustering analyses indicated a close genetic relationship between the Mlabri and the Htin, a group speaking a Tin language.

**Conclusion:**

Our results strongly suggested that the Mlabri share more recent common ancestry with the Htin. We thus provided, to our knowledge, the first genetic evidence that supports the linguistic affinity of Mlabri, and this association between linguistic and genetic classifications could reflect the same past population processes.

## Background

The Mlabri are a hill tribe in northern Thailand, inhabiting a dispersed area along the border with Laos [1,2]. Today, they are a small population of nomadic hunter-gatherers, unusual in a region of almost entirely agricultural economies [3]. The modern population size is estimated at around 300 individuals, with some estimates being as low as 100 [4]. The name Mlabri is a Thai/Lao alteration of the word Mrabri, which appears to derive from a Khmuic term for "people of the forest" - in Khmu, mra means "person" and bri "forest". They are also known locally as Phi Tong Luang or "spirits of the yellow leaves", apparently because they abandon their shelters when the leaves begin to turn yellow with the onset of the dry season.

Little is known about the origins of the Mlabri and most evidence comes from linguistic studies. The Mlabri language is classified as a Khmuic language, a subgroup of the Mon-Khmer language in the Austro-Asiatic language family [5]. The available linguistic evidence suggests that the present-day Mlabri language most likely arose from Tin, a Khmuic language [2,6]. However, so far there is no genetic evidence supporting this idea. A recent study suggested Mlabri was founded recently from an agricultural group, thus representing a typical example of cultural reversion [7]. This work, although very interesting, was criticized for not including any of populations neighboring the Mlabri, such as the Htin, Hmong, and northern Thai. As a result, these authors were unable to demonstrate any similarities in the genetic and linguistic affinity of the Mlabri, and so made little comment on the possible source population(s) from which the Mlabri originated [8].

In this study, we analyzed populations samples from throughout northern Thailand, including the Mlabri as well as several neighboring groups, including the Htin, Hmong, Yao, and other populations speaking Austro-Asiatic and Tai-Kadai languages. Four HapMap population samples, representing Altaic, Sino-Tibetan, Indo-European and Niger-Congo language speakers, were also included in this study. We conducted a genome-wide analysis on these samples using 50K SNPs, to investigate the genetic affinity of the Mlabri, examine the concordance of genetic and linguistic affinities, and further explore probable origin(s) of this enigmatic hunter-gatherer group.

## Results

### Genetic Characteristics of Mlabri

Since this is the first genome-wide genetic study of this enigmatic population, we calculated several population genetic parameters, including SNP diversity, haplotype diversity and linkage disequilibrium (LD).

#### Reduced genetic diversity in the Mlabri

**Figure 1:**
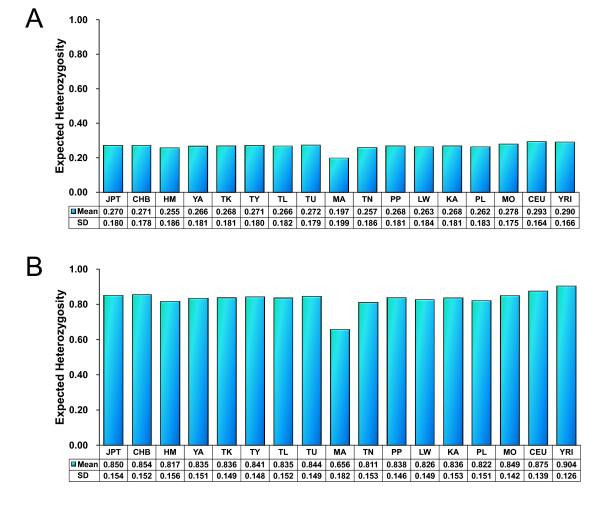
**Heterozygosity in 17 populations**. In table at the bottom of each plot displayed the average and the standard deviation of H_e _in each population sample. The sample information of each population is shown in **Table 1**. **A**: H_e _were calculated from 55,561 SNPs shared by 17 populations. SD denotes standard deviation of the H_e _values across 55,561 SNPs. **B**: H_e _were calculated from haplotypes of 500 kb windows, SD denotes standard deviation of the H_e _values across windows.

Expected heterozygosity for SNPs (HS_e_) were calculated based on allele frequencies of 55,561 SNPs and the results were shown in Figure 1A. The HS_e _in Mlabri (0.197) is lower than that of any of other populations in which HS_e _is at least 0.250 (HM). The expected heterozygosity for haplotypes (HH_e_) were calculated based on haplotypes in 500-kb genomic regions (Methods) and the results are shown in Figure 1B. The HH_e _in Mlabri (0.666) is also much lower than that of any of other populations in which HH_e _is at least 0.820 (TN). The HH_e _comparison obtained from larger size of genomic regions (1 Mb) show the similar results (see Additional file 1, Figure S1). All the above comparisons are statistically significant (t-test, p < 10^-5^).

**Figure 2:**
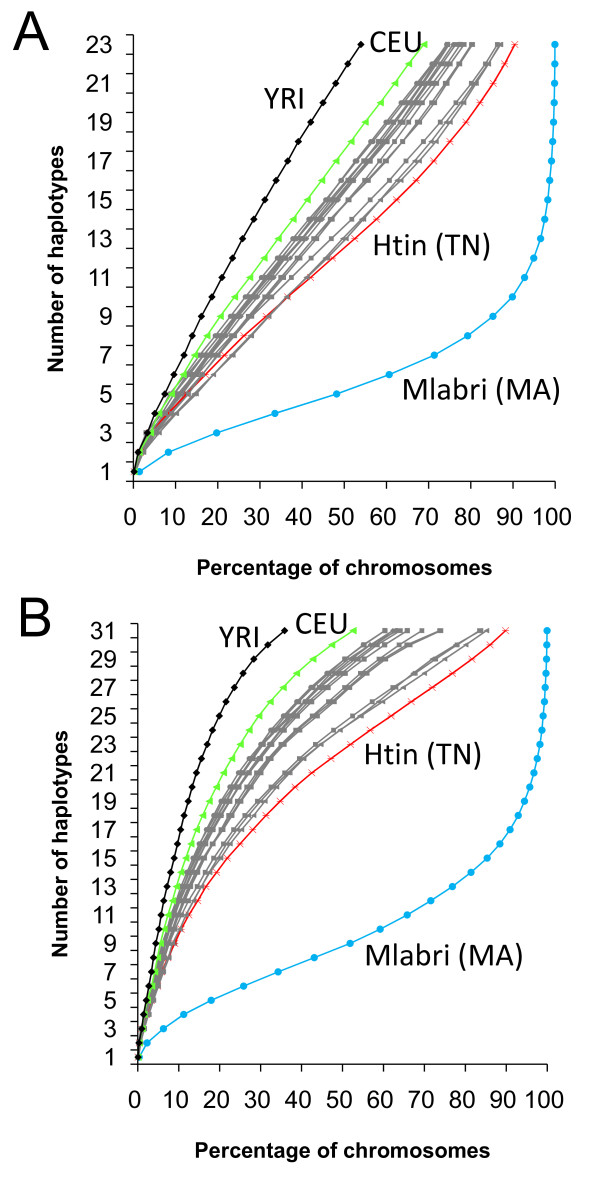
**Number of haplotypes and their cumulative proportion**. **(A) **The number of haplotypes and their cumulative proportion in the genome for 500-Kb sliding windows. **(B) **The number of haplotypes and their cumulative proportion in the genome for 1-Mb sliding windows.

We also compared genetic diversity among populations using the cumulative proportion of the genome given the number of haplotypes (see Methods). The number of haplotypes was estimated for two different window sizes (500-kb or 1-Mb) respectively, with adjustment for sample size difference among populations (see Methods). Again, we found that the genetic diversity was significantly lower in Mlabri than in other populations for both 500-kb segments (Figure 2A) and 1-Mb segments (Figure 2B), respectively. For example, in Mlabri, 99% of the 500 kb segments across the genome carry 17 or less haplotypes in Mlabri, and it is much larger than those in other East Asian populations (52% ~68%), CEU (48%), and YRI (20%).

#### Increased linkage disequilibrium in the Mlabri

**Figure 3:**
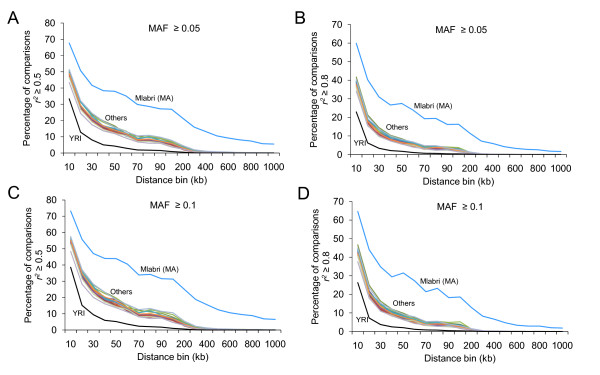
**Decay of linkage disequilibrium (LD) over distance**. LD was measured by pairwise comparison (**A**&**C**, r^2 ^≥ 0.5; **B**&**D**, r^2 ^≥ 0.8) between markers that had minor allele frequency ≥ 0.05 (**A, B**), ≥ 0.1 (**C, D**) and that fell into the same intermarker distance bin. Blue lines denote Mlabri, black lines denote YRI, others include the rest 15 population samples.

The significantly reduced genetic diversity in Mlabri was also reflected by its extent of linkage disequilibrium (LD). We assessed the extent of LD among markers with minor allele frequency (MAF) ≥ 0.05 (Figure 3A, B) and ≥ 0.1 (Figure 3C, D). The LD extended substantially longer in Mlabri than all the other populations, measured as the fraction of SNP pairs with r^2 ^≥ 0.5 (Figure 3A, C) or r^2 ^≥ 0.8 (Figure 3B, D). For marker pairs with moderate LD (r^2 ^≥ 0.5), we observed this fraction to be 1.6- to 12.3-fold higher in Mlabri than in all the other Asian populations for the distance range above 10-kb to 200-kb. For those marker pairs with strong LD (r^2 ^≥ 0.8), the fraction in Mlabri is 2.2- to 31.3-fold higher in Mlabri than in all the other Asian populations, and 6- to 259-fold higher than in YRI. Furthermore, LD of r^2 ^≥ 0.8 extended more than 1 Mb in Mlabri, whereas in all the other populations, such strong LD extended only up to 200 kb.

### Genetic Affinity of Mlabri

The genetic characteristics obtained from above analysis, such as significantly increased LD and extremely reduced haplotype diversity are both consistent with the view from a previous study [7] that the Mlabri were recently founded from a very small number of individuals. The available linguistic evidence suggests that the present-day Mlabri language arose from a Khmuic language, most likely Tin [2,6,7]. To search for the group that gave rise to the founders of Mlabri and to examine if the genetic affinity is consistent with linguistic affinity, we further investigated the genetic relationship of Mlabri and other populations. The rational is that the group with closest genetic relationship with Mlabri, if also consists with linguistic relationship, is most likely the genetic and linguistic founder source.

### Individual-based clustering analysis

**Figure 4:**
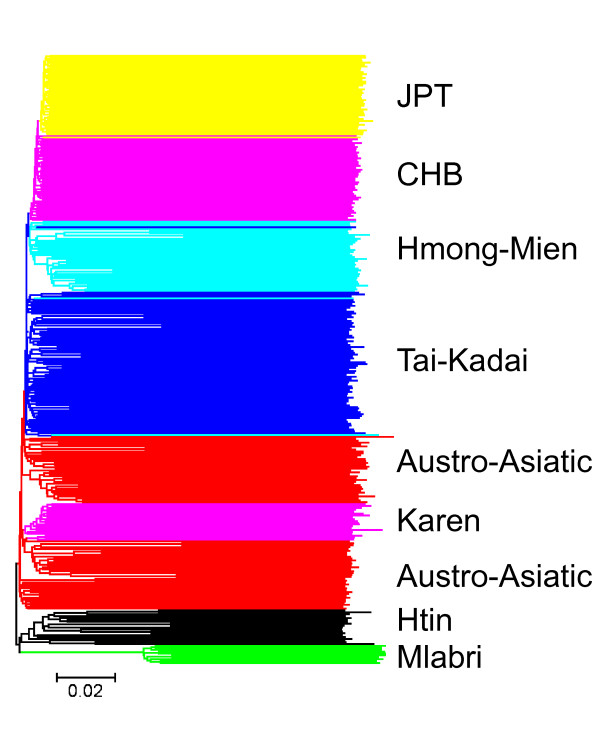
**Relationships among 446 individuals reconstructed using Neighbor-Joining method on a matrix of allele sharing distances (ASDs)**. Pairwise ASD was calculated using 55,561 autosomal SNPs. Individuals are shaded by different colors according to their ethnic or linguistic affiliations.

We first studied the clustering relationships among 446 individuals representing 13 populations in Thailand and the CHB and JPT from the HapMap project (YRI and CEU samples were not included in this analysis). We used an allele sharing distance (ASD) [9] as the genetic distance between individuals and reconstructed an individual tree (Figure 4) using the Neighbor-Joining algorithm [10]. There are several clear clusters on the tree which coincide with individual linguistic or ethnic affiliations, for example, as denoted in Figure 4, JPT, CHB, Hmong-Mien, Tai-Kadai, Austro-Asiatic, Htin and Mlabri. Notably, all the Mlabri and Htin individuals cluster together tightly (100 per cent bootstrap) although there is a bifurcation between clusters of Mlabri and Htin, indicating that the Mlabri have a closer relationship with the Htin than any of other populations studied.

**Figure 5:**
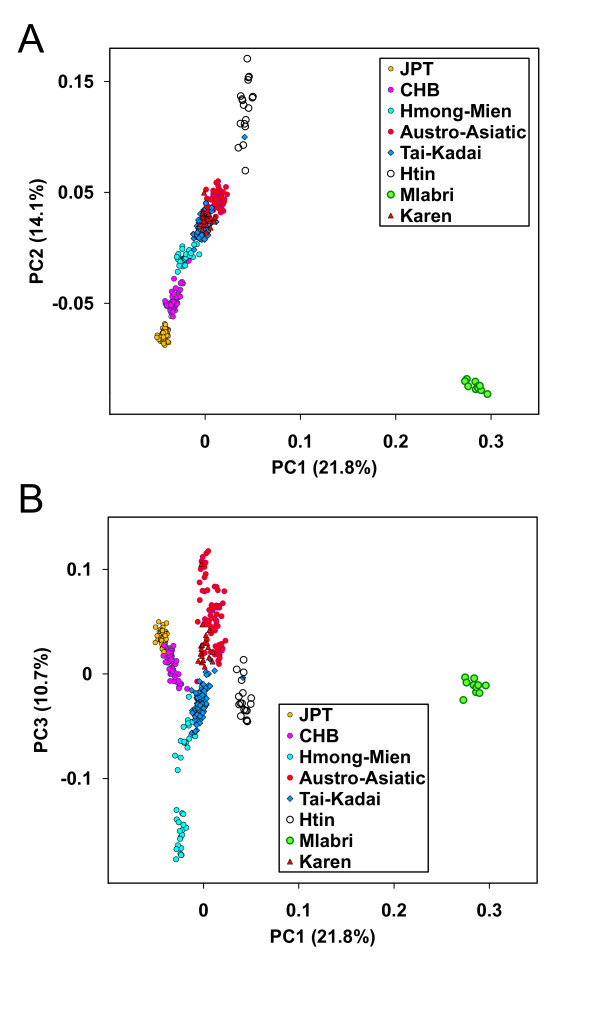
**Plot of Principal Components for 446 individuals representing 15 populations**. Individuals are shaded by different colors according to their predefined population affiliations, and the legend is displayed on the lower right of the plot. **A**: plot of the first two principle components. **B**: plot of the first and the third principle components.

The above clustering relationships among individuals were also confirmed by principal components analysis (PCA) at the individual level [11]. As shown in a 2-dimentional plot of first two PCs (Figure 5A), individuals tend to cluster with other members of their linguistic or ethnic affiliations. Again, Mlabri showed a closer relationship with the Htin for PC1, which explains 21.8% of variation represented by the first ten PCs. The closer relationship between Mlabri and Htin is even more pronounced in the 2-dimentional plot of PC1 and PC3 (Figure 5B).

**Figure 6:**
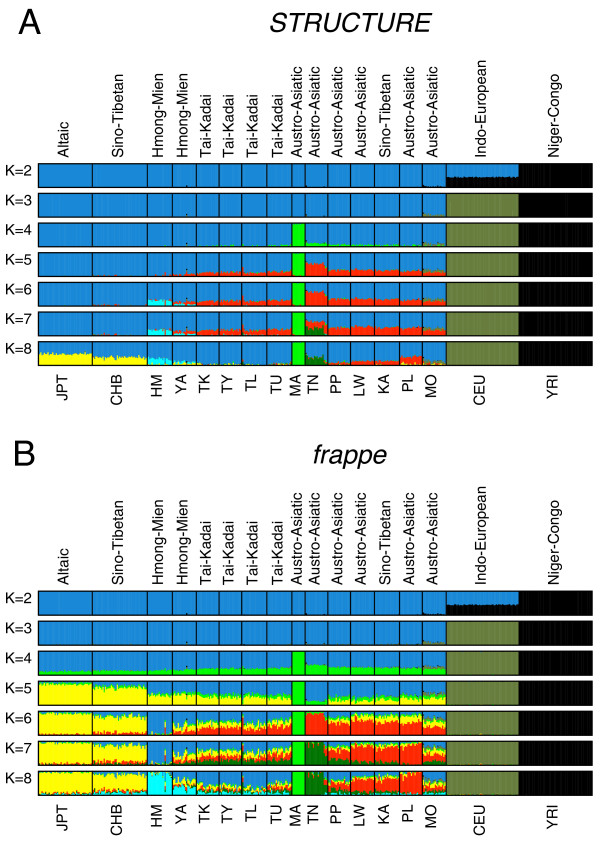
**Estimated population structure**. Each colored vertical line represents an individual that is assigned proportionally to one of the K clusters with the proportions represented by the relative lengths of the K different colors. Black lines separate individuals of different populations. Populations are labeled below the figure with the same convention shown in Table 1 and Figure 1. Left plot: population structure inferred by STRUCTURE; right plot: population structure inferred by frappe. For both STRUCTURE and frappe results, the figure shown for a given K is based on the highest probability run of ten runs at that K.

Since the closer relationship between Mlabri and Htin could be due to recent gene flow from Htin to Mlabri or vice versa, we further performed Bayesian cluster analysis as implemented in the STRUCTURE algorithm [12] to examine the ancestry of each person. This analysis considers each person's genome as having originated from K ancestral, but unobserved, populations whose contributions are described by K coefficients that sum to 1 for each individual [13]. Individuals are posited to derive from an arbitrary number of ancestral populations, denoted by K. We ran STRUCTURE from K = 2 to K = 18, with results at K = 8 showing the greatest posterior probability (see Additional file 2, Figure S2). Estimated individual membership fractions in K genetic clusters are shown in Figure 6A. At K = 3, the three clusters correspond with Asian, European and African ancestry, respectively. At K = 4, the new cluster corresponds to a Mlabri specific component, which is exclusively shared by all Mlabri individuals with 100 percent membership fractions and this pattern persisted for all choices of K>3. Similar analyses were also performed using the program frappe [14] which implements a maximum likelihood method. The results obtained from frappe (Figure 6B) showed a general concordance with that of STRUCTURE; but slight differences were also observed, such as the order with which new clusters emerge at K = 5 and K = 6, and the estimated individual membership fractions for all K>3. Notably, both analyses showed that all Asian populations shared some proportion of the major Mlabri component at K = 4 and K = 5. However, this sharing pattern, unless it is an artifact, is more likely to be explained by shared common ancestry rather than recent gene flow, because it appears highly unlikely that the Mlabri received (or contributed) nearly identical amounts of gene flow from (or to) all Asian populations, and with similar proportions, in every instance. Therefore, the close relationship between Mlabri and Htin is most likely the result of a considerable degree of common ancestry.

### Population- and component-based clustering analyses

**Figure 7:**
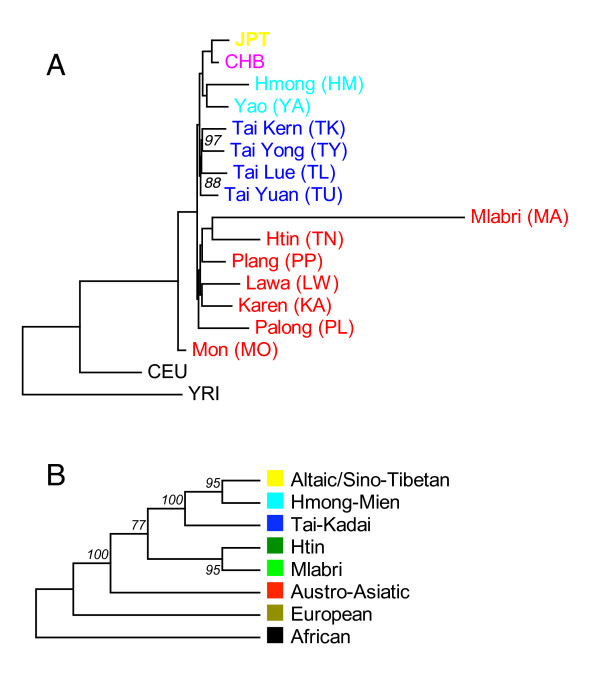
**Maximum likelihood tree of populations and components**. **A**: Maximum likelihood tree of 17 populations, bootstrap values obtained by sampling 55,561 SNPs 100 times with replacements, only values less than 100 are shown. Colored population names (IDs) help recognize their linguistic affinities, yellow: Altaic, magenta: Sino-Tibetan, light-blue: Tai-Kadai, red: Austro-Asiatic. **B**: Maximum likelihood tree of components inferred from STRUCTURE analysis (K = 8) Bootstrap values for **B **was obtained by randomly sampling cluster frequencies 100 times from STRUCTURE results.

Because the analyses discussed above were all consistent in showing that individuals from the same population cluster together, it is meaningful to evaluate the genetic relationships among populations. A maximum likelihood tree of populations [15], based on 55,561 SNPs showed that Mlabri (MA) and Htin (TN) have the closest relationship, and this topology was supported by 100% of bootstrap replicates (Figure 7A).

However, the Htin showed signs of admixture in both STRUCTURE and frappe analyses (Figure 6A, B). This raised the concern that whether the close relationship between Mlabri and Htin was confounded by external immigrants from other populations, given that about half of components of Htin are also found in both Austro-Asiatic and Tai-Kadai populations at Ks>4 in STRUCTURE results (Figure 6A). We thus further investigated this potential confounding effect by reconstructing the phylogenetic relationships of those clusters inferred from STRUCTURE and frappe (referred to as the "component tree"). The rationale is that the component tree, given the statistical independence of the components, should reveal an evolutionary history that is less perturbed by recent gene flow and admixture than is a population phylogeny. At K = 8, both STRUCTURE and frappe identified a cluster predominant in the Htin, and with each of the other seven clusters easily associated with a predominant linguistic or ethnic group. We therefore refer to the eight clusters (or components) by their representative linguistic or ethnic group as follows: Altaic/Sino-Tibetan, Hmong-Mien, Tai-Kadai, Austro-Asiatic, Mlabri, Htin, European and African. The component tree was reconstructed based on allele frequencies in each cluster inferred from the STRUCTURE analysis (Figure 7B). We found that the Mlabri specific and Htin specific component clustered tightly on the tree (supported by 100% of bootstrap replicates), strongly indicating once again that the Mlabri share a more recent ancestry with the Htin than with any other group in our sample.

## Discussion

In this study, we analyzed genome-wide SNP data on the Mlabri, as well as several neighboring populations and HapMap population samples. The Mlabri population shows several substantial differences from the other populations: significantly increased LD, extremely reduced haplotype diversity and small effective population size (29), all of which are consistent with the view that the Mlabri were recently founded from a very small number of individuals of an agricultural group but subsequently adopted their current hunting-gathering lifestyle, as proposed by a recent study based primarily on mtDNA and Y chromosome data [7]. Although an alternative scenario could also explain the above genetic characteristics of Mlabri, i.e. the Mlabri are an ancient hunter-gatherer group and maintain their hunting-gathering lifestyle from the very beginning but experienced a severe bottleneck event in the history, the results from the clustering analyses do not favor this scenario. If the Mlabri are an ancient hunter-gatherer group, we expect Mlabri is outside of the clade of all Asian populations and close to the root of Asian clade, but Mlabri is actually inside of Asian clade with Austro-Asiatic group outside on both population tree (Figure 7A) and component trees (Figure 7B, C) where no signal of admixture was found have disturbed tree topology.

Both model-free and model-based clustering analyses strongly suggest that the Mlabri share a degree of common ancestry with the Htin, a group speaking Tin language. In this case -- as is the general rule in many human populations -- the genetic affinity of these populations is consistent with its linguistic affinity. This result, to our knowledge, is the first genetic evidence supporting the linguistic affinity of the Mlabri and Tin languages. Cavalli-Sforza and colleagues showed an apparent congruence between linguistic phyla and genetic clusters, and they proposed that this congruence indicates "considerable parallelism between genetic and linguistic evolution" [16]. Subsequent studies using diverse scales and methodologies have found variable degrees of association between linguistic and genetic classifications [17-22]. Some typical examples of exceptions are populations with language replacement [23-26] or recent admixture between divergent populations [27,28]. However, human genetic and linguistic diversity have been proposed to be generally correlated, either through a direct link, whereby linguistic and genetic affiliations reflect the same past population processes, or an indirect one, where the evolution of the two types of diversity is independent but conditioned by the same geographic factors [29].

Hunting and gathering was presumably the subsistence strategy employed by human societies for more than two million years, until the end of the Mesolithic period. Contemporary hunter-gatherer groups are often thought to serve as models of an ancient lifestyle that was typical of human populations prior to the development of agriculture. However, there has been complex interaction between hunter-gatherers and non-hunter-gatherers for millennia. There are contemporary hunter-gatherer peoples who, after contact with other societies, continue their ways of life with very little external influence. There are also contemporary groups usually identified as hunter-gatherers do not have a continuous history of hunting and gathering, and in many cases their ancestors were agriculturalists and/or pastoralists who were pushed into marginal areas as a result of migrations, economic exploitation, and/or violent conflict [30]. Our current data are not sufficient to distinguish the two scenarios, but in case cultural reversion occurred in the history of Mlabri, the Htin is most likely the source population from which the Mlabri genetically originated. The Htin samples in this study speak Mal language, represent only one of the two varieties (Mal and Prai) of Tin language [31,32], it is possible to further determine which variety the Mlabri language originated from by comparing the genetic relationships between the Mlabri and populations speak the two Tin varieties, although such evidence is indirect and would only make sense when the assumption hold that the genetic origin of the Mlabri was not earlier than the divergence of the two language varieties and there was no language replacement.

## Conclusions

In summary, our results strongly suggested that the Mlabri share more recent common ancestry with the Htin, a group speaking a Tin language. This result, to our knowledge, is the first genetic evidence supporting the linguistic affinity of the Mlabri and Tin languages. We proposed that Htin is most likely the source population from which the Mlabri genetically originated in case cultural reversion occurred in the history of Mlabri.

## Methods

### Populations and Samples

Samples from the Mlabri as well as other 12 populations were collected in Thailand. The sample information, including sample size, ethnic and linguistic information is shown in Table 1, and the sampling locations are shown in Additional file 3, Figure S3. These samples were also described previously [33]. In this study, eight Mlabri samples were not included because they were identified as close relatives (IBD > 0.2) of one of the rest samples. Four population samples (60 YRI, Yoruba from Ibadan, Nigeria; 60 CEU, Utah residents with ancestry from northern and western Europe; 45 CHB, Han Chinese in Beijing; and 44 JPT, Japanese in Tokyo) obtained from the database of the International HapMap Project [34] were also included in this study.

**Table 1:** Information of population samples.

Population ID	Ethnicity	Language family	Language	Sample-size
JPT	Japanese	Altaic	Japanese	44
CHB	Han	Sino-Tibetan	Chinese	45
HM	Hmong	Hmong-Mien	Hmong	20
YA	Yao	Hmong-Mien	Iu-Mien	19
TL	Tai Lue	Tai-Kadai	Lue	20
TY	Tai Yong	Tai-Kadai	Yong	18
TK	Tai Kern	Tai-Kadai	Kern	18
TU	Tai Yuan	Tai-Kadai	Yuan	20
PL	Palong	Austro-Asiatic	Palong	18
KA	Karen	Sino-Tibetan	Karen	20
LW	Lawa	Austro-Asiatic	Lawa	19
PP	Plang	Austro-Asiatic	Blang	18
TN	Htin	Austro-Asiatic	Mal	18
MA	Mlabri	Austro-Asiatic	Mlabri	18
MO	Mon	Austro-Asiatic	Mon	19
CEU	European	Indo-European	English	60
YRI	Yoruba	Niger-Congo	Yoruba	60

### Data Sets

Genotype data of 13 Thailand population samples generated using Affymetrix Genechip Human Mapping 50K Xba array were obtained from the Pan-Asian SNP Initiative [33]. Detailed information about data filtration and data quality control was described elsewhere [33]. Genotypes of 60 YRI, 60 CEU, 45 CHB and 44 JPT samples were obtained from the International HapMap Project [34-36] (HapMap public released #23a, 2008-04-01). Most of the analyses in this study used the markers that genotyped in both PanAsia project and HapMap project, including 55,561 autosomal SNPs shared by 13 Thailand population samples and 4 HapMap population samples.

### Statistical analysis

#### Haplotype inference

Haplotypes of 22 autosomes were inferred for each individual from its genotypes with fastPHASE [37] version 1.2. "Population labels" were applied during the model fitting procedure to enhance accuracy. The number of haplotype clusters was set to 20, the number of random starts of the EM algorithm (-T) was set to 20, and the number of iterations of EM algorithm (-C) was set to 50. This analysis was used to generate a "best guess" estimate of the true underlying patterns of haplotype structure [37]. We run fastPHASE for 55,561 SNPs shared by 17 populations, and only unrelated individuals were included.

#### SNP heterozygosity

Heterozygosity for each SNP (HS_e_) was calculated based on allele frequencies.

#### Haplotype heterozygosity

To calculate heterozygosity for haplotypes (HH_e_), the genome was divided into 500-kb regions, with each region having roughly 14 SNPs. HH_e _were calculated for each region using haplotype frequencies [38]. Considering the substantial variation of recombination across human genome [39,40], we adopted a slide window strategy and let the sliding window move 100 kb each time. For each population, HH_e _were averaged over all windows.

#### Number of haplotypes and its cumulative proportion of the genome

The number of haplotypes was obtained by counting the number of haplotypes for a given window size, i.e. 500-kb or 1-Mb, respectively, for each population. The same sliding-window scheme as mentioned before was employed. Since this measurement could be affected by sample size, we sampled 36 chromosomes (equal to the sample size of Mlabri) without replacement in each population. Note that Mlabri has the smallest sample size in all the populations studied. For a population with sample size larger than 36 chromosomes, the sampling was repeated 100 times for each segment and the average of the number of haplotypes of all replications was taken as the number of haplotypes.

The cumulative proportion given a number of haplotypes was obtained by estimating the proportion of the sliding-windows across the genome carrying equal or less haplotypes.

#### LD calculation

Linkage disequilibrium (LD) between SNPs were measured using r^2 ^following Hill and Weir [41] and calculated from haplotype data.

#### Principal component analysis for individuals

Principal component analysis (PCA) was performed at individual level using EIGENSOFT version 2.0 [42].

#### Genetic distance for individuals

We used an allele sharing distance (ASD) [9,43] as a measure of genetic distance between individuals and a 454 × 454 inter-individual genetic distance matrix was generated according to genotypes of 55,561 autosomal SNPs.

#### Tree reconstruction

The tree of individuals was reconstructed based on ASD distance and using Neighbor-Joining algorithm [10] with the Molecular Evolutionary Genetics Analysis software package (MEGA version 4.0) [44]. Trees of populations as well as components were reconstructed using maximum likelihood method [15] with CONTML program in PHYLIP package [45].

#### STRUCTURE analysis

Ancestry of each person was inferred using a Bayesian cluster analysis as implemented in the STRUCTURE program [12,46]. We ran STRUCTURE from K = 2 to K = 18 and repeated 10 times for each single K. All STRUCTURE runs used 20,000 iterations after a burn-in of length 30,000, with the admixture model and assuming that allele frequencies were correlated [46].

#### frappe analysis

The program frappe [14] implements a maximum likelihood method to infer genetic ancestry of each individual. As in STRUCTURE analysis, this analysis considers each person's genome as having originated from K ancestral, but unobserved, populations whose contributions are described by K coefficients that sum to 1 for each individual [13]. The program was run for 10,000 iterations from K = 2 to 18 and repeated 10 times for each single K.

## Authors' contributions

SX, DK, MS, and LJ conceived and designed the study. JK contributed to the Tai-Kadai sample collection. MS and MS performed the technical work on SNP genotyping and QC procedures for all population samples from Thailand. SX collected data and performed the analysis. SX and LJ wrote the paper, to which all authors contributed. All authors read and approved the final manuscript.

## Supplementary Material

Additional file 1 **Contains Figure S1 - Haplotype heterozygosity (HH_e_) in 17 populations.** In table at the bottom of each plot displayed the average and the standard deviation of HH_e _in each population sample. The sample information of each population is shown in Table 1. HH_e _were calculated from haplotypes of 1-Mb windows, SD denotes standard deviation of the HH_e _values across windows. Click here for file

Additional file 2 **Contains Figure S3 - Probability Estimations for the Number of Clusters, with Ten Repeats for Each K.** The ordinate shows the Ln probability corresponding to the number of clusters (K) shown on the abscissa. A: showing maximal probability estimation of ten runs at each K (from K = 2 to K = 9); B: showing probability estimation at K = 2 to K = 18 in all ten runs. Click here for file

Additional file 3 **Contains Figure S2 - Geographical distribution of Thailand population samples.** Red dots on the map indicated sampling locations. Information of population IDs can be found in Table 1. Click here for file
