# Supplementary figures and images for: Genetic evidence supports linguistic affinity of Mlabri - a hunter-gatherer group in Thailand
Source: BMC Genet. 2010 Mar 19;11:18. doi: 10.1186/1471-2156-11-18 (PMC2858090; doi:10.1186/1471-2156-11-18)

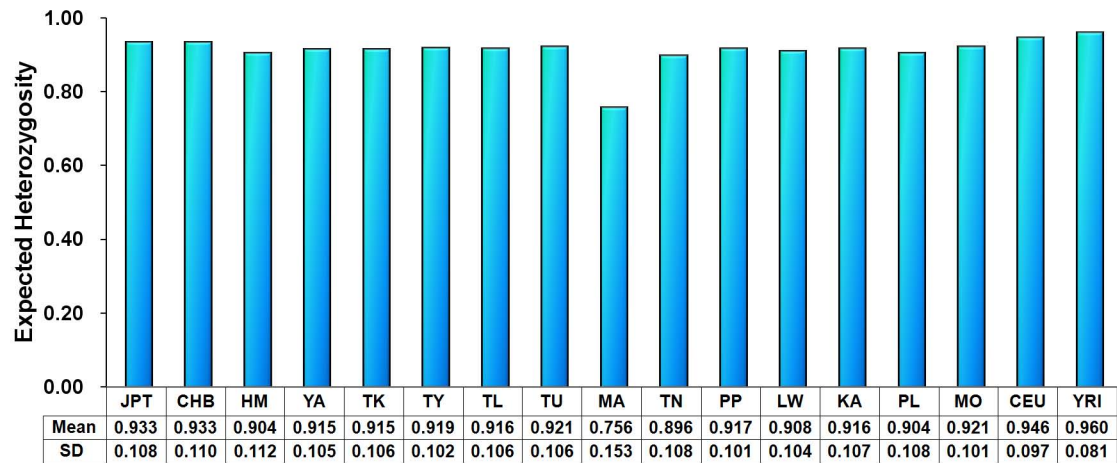

Supplement: Additional file 1 — Contains Figure S1 - Haplotype heterozygosity (HHe) in 17 populations. In table at the bottom of each plot displayed the average and the standard deviation of HHe in each population sample. The sample information of each population is shown in Table 1. HHe were calculated from haplotypes of 1-Mb windows, SD denotes standard deviation of the HHe values across windows. [file 1471-2156-11-18-S1.PDF]

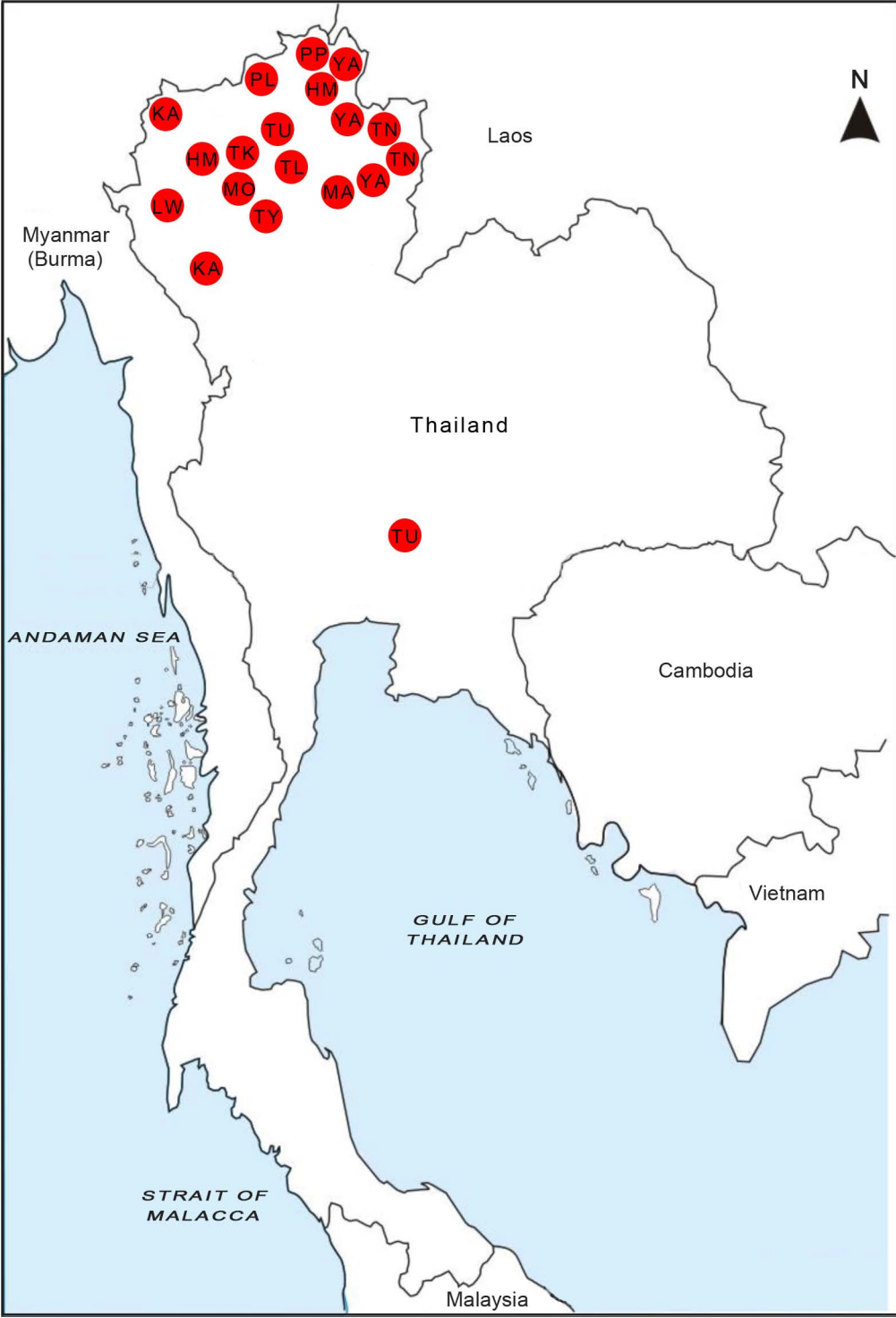

Supplement: Additional file 3 — Contains Figure S2 - Geographical distribution of Thailand population samples. Red dots on the map indicated sampling locations. Information of population IDs can be found in Table 1. [file 1471-2156-11-18-S3.PDF]
