# Supplementary material for: Genetic evidence supports linguistic affinity of Mlabri - a hunter-gatherer group in Thailand
Source: BMC Genet. 2010 Mar 19;11:18. doi: 10.1186/1471-2156-11-18 (PMC2858090; doi:10.1186/1471-2156-11-18)
Supplement: Additional file 2 — Contains Figure S3 - Probability Estimations for the Number of Clusters, with Ten Repeats for Each K. The ordinate shows the Ln probability corresponding to the number of clusters (K) shown on the abscissa. A: showing maximal probability estimation of ten runs at each K (from K = 2 to K = 9); B: showing probability estimation at K = 2 to K = 18 in all ten runs. [file 1471-2156-11-18-S2.PDF]

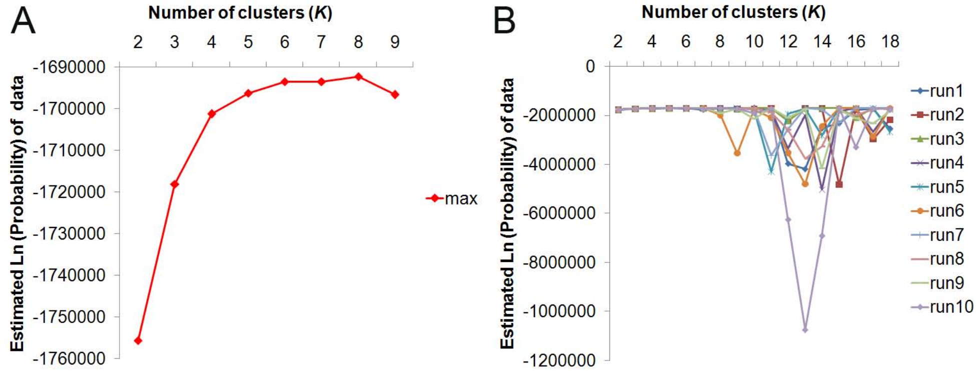

A
Number of clusters (K)
2
3
4
5
6
7
8
9
-1690000
-1700000
-1710000
-1720000
-1730000
-1740000
-1750000
-1760000
Estimated Ln (Probability) of data
max
B
Number of clusters (K)
2
4
6
8
10
12
14
16
18
0
-2000000
-4000000
-6000000
-8000000
-1000000
-1200000
Estimated Ln (Probability) of data
run1
run2
run3
run4
run5
run6
run7
run8
run9
run10
